# Under-expression of CK2β subunit in ccRCC represents a complementary biomarker of p-STAT3 Ser727 that correlates with patient survival

**DOI:** 10.18632/oncotarget.23422

**Published:** 2017-12-19

**Authors:** Jordi Vilardell, Estefania Alcaraz, Eduard Sarró, Enric Trilla, Thaïs Cuadros, Inés de Torres, Maria Plana, Santiago Ramón y Cajal, Lorenzo A. Pinna, Maria Ruzzene, Juan Morote, Anna Meseguer, Emilio Itarte

**Affiliations:** ^1^ Departament de Bioquímica i Biologia Molecular, Unitat de Bioquímica, Facultat de Biociències, Universitat Autònoma de Barcelona, Bellaterra, Barcelona, Spain; ^2^ Fisiopatología Renal, CIBBIM, VHIR, Barcelona, Spain; ^3^ Servicio de Urología, Hospital Vall d’Hebrón, Barcelona, Spain; ^4^ Servicio de Anatomía Patológica, Hospital Vall d’Hebrón, Barcelona, Spain; ^5^ Centro de Investigación Biomédica en Red en Bioingeniería, Biomateriales y Nanomedicina (CIBER-BBN), Madrid, Spain; ^6^ Spanish Biomedical Research Network Centre in Oncology (CIBERONC), Barcelona, Spain; ^7^ Department of Biomedical Sciences and CNR Institute of Neuroscience, University of Padova, Padova, Italy; ^8^ Departament de Bioquimica i Biologia Molecular, Unitat de Bioquímica de Medicina, Universitat Autònoma de Barcelona, Bellaterra, Barcelona, Spain; ^9^ Instituto Reina Sofía de Investigación Nefrológica, Fundación Renal Íñigo Álvarez de Toledo, Madrid, Spain; ^10^ Red de Investigación Renal (REDINREN), Barcelona, Spain

**Keywords:** protein kinase CK2, clear cell renal cell carcinoma (ccRCC), epithelial-to-mesenchymal transition (EMT), STAT3, patient outcome

## Abstract

Clear cell renal cell carcinoma (ccRCC) is the most common and aggressive subtype of renal cancer. STAT3 pathway is altered in these tumors and p-STAT3 Ser727 is an independent prognostic factor for ccRCC. Protein kinase CK2 is altered in different types of tumors and overexpression of CK2α is considered predictive of bad prognosis and metastatic risk. CK2 subunits analyses in ccRCC samples showed increased CK2α/α’ nuclear content in all cases, but decreased cytosolic CK2β (CK2βcyt) levels in the more advanced tumors. Stable downregulation of CK2β in renal proximal tubular (HK-2) and clear cell adenocarcinoma (786-O) cells triggered changes in E-cadherin, vimentin and Snail1 protein levels indicative of epithelial-to-mesenchymal transition (EMT), and increased HIF-α. Moreover, CK2β was required in order to observe STAT3 Ser727 phosphorylation in HK-2 but not in 786-O cells.

We also observed that CK2β improved the prognostic value of p-STAT3 Ser727, as CK2βcyt>41 (median value) discriminates patients free of disease for a period of 10 years upon surgery, from those with CK2βcyt<41, when p-STAT3 Ser727levels are low.

We conclude that CK2β down-regulation might represent a mechanism to support EMT and angiogenesis and that CK2βcyt levels are instrumental to refine prognosis of ccRCC patients with low p-STAT3 Ser727 levels.

## INTRODUCTION

Renal cell carcinoma (RCC) is the sixteenth most common cause of death from cancer worldwide and its incidence and mortality rates have been increasing in many countries in the last years [1, 2]. The clear cell RCC (ccRCC) is the most frequent RCC subtype, comprising 80–90% of the malignant renal tumors in adults [3]. Originated from renal cells of the proximal convoluted tubule, ccRCC is characterized by the absence of symptomatology until advanced stages of the disease, what is associated with a poor prognosis [4]. ccRCC is highly resistant to radiotherapy and to conventional cytotoxic chemotherapy. Albeit novel targeted therapies have been implemented since 2008, treatment resistance remains a problem for treatment of metastatic patients what makes surgical resection the most common treatment for localized disease [3]. However, approximately one-third of the patients have metastatic disease detected at presentation, and 20 to 30% of all ccRCC patients undergoing nephrectomy exhibit local or distant disease recurrence during the follow-up. Patients with distant metastases have a poor prognosis and their 5-year survival rate is reduced to around 10%.

Epithelial-to-mesenchymal transition (EMT), a well characterized process in embryogenesis and wound healing, has also been linked to tumor progression and metastasis in a wide number of carcinomas including RCC [5, 6]. Several signaling pathways, including hypoxia (HIF1/2) pathway are inducers of the EMT phenotype [7] and it is well known that ccRCCs display deregulation of HIF1/2 pathway [8, 9]. The signal transducer and activator of transcription-3 (STAT3) pathway is also deregulated in human RCC [10] and STAT3 has been reported to act as a potent regulator of HIF-1α and HIF-2α expression [11] and as a modulator of HIF-1-mediated response [12]. STAT3 activation is reflected through its phosphorylation at Tyr705 and Ser727 residues, both of which are increased in ccRCC tumors. In fact, we have recently shown that p-STAT3 Ser727 is an independent prognostic factor for ccRCC patients [13].

Different reports have shown that protein kinase CK2 is involved in HIF-1α activation and stabilization [14–16]. Moreover, CK2 activity is necessary for STAT3 activation by IL-6 family cytokines [17] and cell treatment with CK2 inhibitors affect STAT3 phosphorylation on Ser727 [18, 19]. CK2 is a constitutively active serine/threonine kinase present in all eukaryotic organisms which acts on a large number of protein substrates involved in a plethora of cellular functions [20]. In mammals, CK2 is composed of two catalytic (CK2α and CK2α’) and a dimer of regulatory subunits (CK2β) giving rise to different heterotetrameric forms of the holoenzyme (α_2_β_2_, αα’β_2_ and α’_2_β_2_) [21–23]. The CK2α and CK2α’ show high similarity in their structural and enzymatic characteristics. Besides its central role in assembling CK2 holoenzyme, CK2β also plays an essential role in the aggregation of CK2 holoenzyme into less active/inactive polymeric assemblies which support an autoinhibitory mechanism of regulation of CK2 [22]. In addition, CK2β helps to discriminate between protein substrates whose phosphorylation is specifically catalyzed by either the free catalytic subunits or CK2 holoenzyme. Interestingly, CK2 holoenzyme is required to phosphorylate snail1 what primes its subsequent phosphorylation by GSK3β, a process that targets it for degradation [24]. Moreover, downregulation of CK2β was enough to stabilize snail1 and to induce an EMT-like phenotype in normal human breast epithelial cells (MCF10A cells) [24].

Increased expression of CK2 catalytic subunits has been shown to occur in different types of human tumors, including renal carcinoma [25–27], and overexpression of both CK2α and CK2α’ is considered as predictive of bad prognostic and metastatic risk [27–33]. The information concerning CK2β status in cancer is rather scarce. A preliminary report indicated that in human renal carcinoma the regulatory CK2β subunit increased even in higher amounts than the catalytic CK2α subunit [25]. In contrast, a recent report using a set of fifteen human renal cell carcinoma cell samples analyzed by western blot detected decreased CK2β levels in some of those samples [26]. However, the correlation between CK2β protein levels and the clinicopathological characteristics of the RCC remains unexplored.

In the present work we aimed to analyze the potential correlation of CK2 subunits with the clinicopathological characteristics of ccRCC tumors and patient survival as well as their impact in HIF expression and STAT3 phosphorylation in human renal cell lines. The results obtained in our work indicate that in ccRCC, CK2 shows not only alterations in the expression of the two catalytic (CK2α’/CK2α) subunits but also in the regulatory CK2β subunit. Moreover, the stable silencing of CK2β in human renal cell lines increases HIFα expression and affects STAT3 phosphorylation. In addition, our results show that cytosolic CK2β levels help to refine the prognostic value of pSer727-STAT3 in ccRCC patients.

## RESULTS

### The expression of CK2 subunits shows asymmetric changes in ccRCC tumor progression

**Figure 1:**
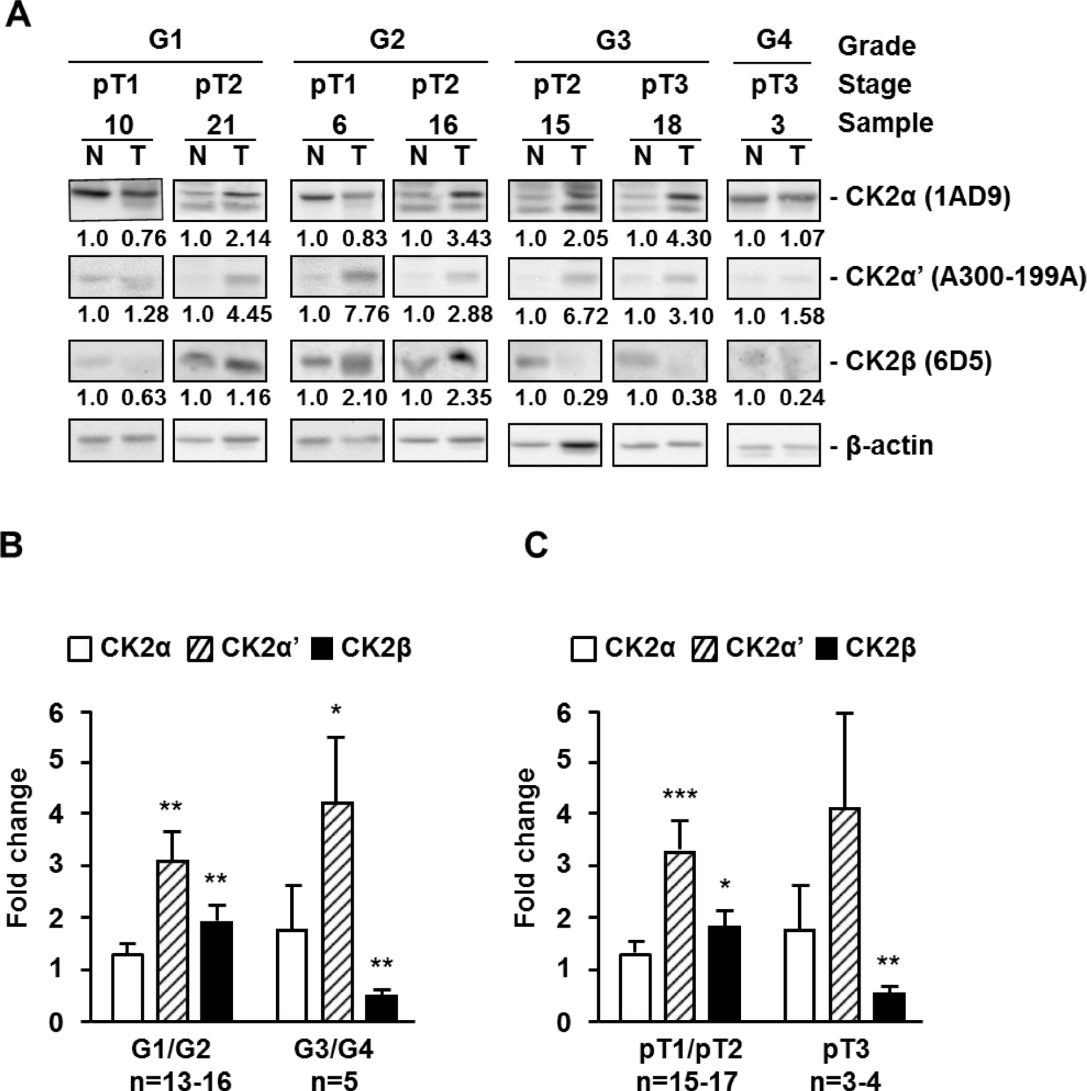
Protein kinase CK2 subunits expression in different extracts of human ccRCC biopsies (**A**) Western blot analysis of CK2α, CK2α’, CK2β and β-actin expression levels in tissue extracts of representative tumor (T) and unaffected normal (N) kidney counterpart samples from ccRCC patients. The type of antibody used to detect each CK2 subunit is indicated in parenthesis. Samples are classified by their grade (G1 to G4) and tumor stage (pT1 to pT3). (**B**), (**C**) Expression ratios of CK2 subunits between tumor and unaffected normal kidney counterpart samples from ccRCC patients, classified by their Fuhrman nuclear grade (B) or their tumor stage (C). Bar graphs show the fold change in CK2α, CK2α’ and CK2β subunit expression in tumors respect to normal kidney counterpart (T/N ratios) after normalization to their β-actin content. Data are represented as mean +/- SEM. Values were analyzed by Student’s *t*-test. (^*^), (^**^), (^***^) denote *p <* 0.05, *p <* 0.01 and *p <* 0.001, respectively. Non-significant differences are not indicated.

A preliminary analysis by western blot of CK2 subunits in a set of frozen tissue samples from patients with ccRCC showed that they did not follow similar patterns along ccRCC tumor progression (Figure 1, Supplementary Figure 1 and Supplementary Table 1). CK2α (1AD9 antibody) and CK2α’ mean values increased in tumor samples of low Fuhrman grade tumors (G1/G2) with respect to normal tissue (set up as fold change of 1) and the increases were even more marked in the G3/G4 group. The CK2α’ values in the G3/G4 tumors were dispersed and the number of tumors too low to reach statistical significance when compared with values observed in G1/G2 tumors. The values shown by the tumors (G1/G2 or G3/G4) were statistically significant respect to values found in normal tissues but they did not reach statistical significance when compared between different tumor groups. On the other hand, CK2β subunit (6D5 antibody) increased in the G1/G2 group but decreased in the more advanced tumors (G3/G4). A similar trend was detected when the samples were combined according to their tumor stage in pT1/pT2 and pT3 groups. Bands detected for CK2α, CK2α’ and CK2β in the immunoblots of Figure 1 have been also found in the 786-O cell line (Supplementary Figure 1). It is likely that extra bands detected in western blots with the anti-CK2α (1AD9 antibody) antibody correspond to proteolytic CK2α products.

In order to use a larger number of ccRCC tumor samples, we decided to evaluate CK2α/α’ and CK2β subunits by immunohistochemistry in tissue microarrays (TMA) including tumor and normal counterpart renal tissue samples and the results were correlated with tumor characteristics. The TMA used included 98 ccRCC patients with the following characteristics: 57.1% men and 42.9% women, median age: 64 years range: 25 to 86. Tumor was right side in 59.1% cases and left in 40.8% cases. Incidental presentation was found in 52.5% patients and 46.9% were symptomatic. 87.9% presented with localized tumors and 11.1% with metastasis. 92.9% patients underwent radical nephrectomy, while 6 underwent nephron-sparing surgery. Median tumor size was 6.4 cm (range 1.5 to 16). Fuhrman grade was I (G1) in 20.4%, II (G2) in 41.8%, III (G3) in 24.4% and IV (G4) in 13.2%. The most frequently observed pT stages were pT1a in 26.5% and pT1b in 27.5%. Lympho-vascular invasion was present in only 5.1% of patients. Finally, UICC risk group was I in 72.4 % and II in 27.5 % of patients studied.

**Figure 2:**
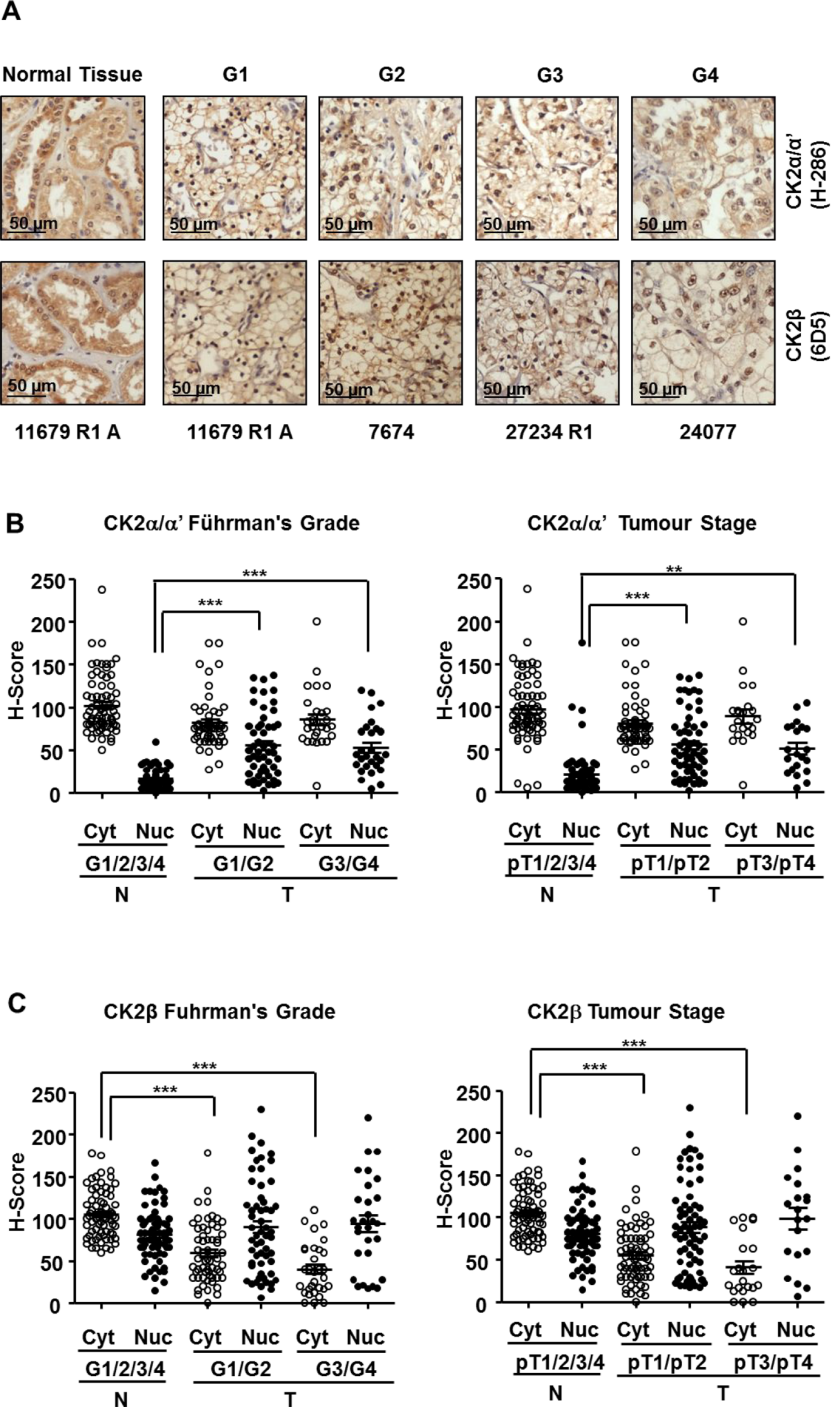
Expression of CK2 subunits in the 98 ccRCC samples embedded in Tissue Microarrays (TMAs) (**A**) CK2α and CK2β levels in tumor tissue samples classified according their nuclear Fuhrman Grade. The pictures correspond to representative samples from biopsies of patients affected by ccRCC with different Fuhrman grades (G1 to G4) as well as a sample of unaffected normal kidney counterpart out of the 98 samples included in the TMA. CK2 subunits (brown) were detected by a colorimetric method and the samples were counterstained by haematoxylin as was indicated in materials and methods. (**B**), (**C**) Scatter plot of CK2α and CK2β expression levels in the nuclei (Nuc) and cytoplasm (Cyt) of the 98 ccRCC samples included in TMAs classified by their Fuhrman nuclear grade (G1/G2, G3/G4) (left hand panels) and stage (pT1/pT2, pT3/pT4) (right hand panels). The samples were evaluated using the H-score semi-quantitative method described previously. H-score values obtained were analyzed by one-way ANOVA using the Bonferroni’s correction algorithm. (^**^), (^***^) denote *p <* 0.01 and *p <* 0.001, respectively. Non-significant differences are not indicated.

Representative images of ccRCC tumors and unaffected normal kidney counterparts, stained with specific antibodies against CK2α and CK2β, are shown in Figure 2. The anti-CK2α/α’ (H-286) and anti-CK2β (6D5) antibodies have been used in previous IHC studies [29, 34, 35]. The specificity of the anti-CK2β antibody (6D5) was also assessed by antibody depletion experiments (Supplementary Figure 2). Positive cytosolic and nuclear staining for CK2α/α’ and CK2β was detected in renal tubular cells in normal counterpart renal tissue (Figure 2A). CK2α/α’ staining in tumor core samples tended to show a prominent nuclear staining (Figure 2A). When analyzing the TMA results according to the tumor Fuhrman grade, we observed that the mean H-Score values for nuclear CK2α/α’ staining were significantly higher in tumors than in normal counterpart renal tissue whereas cytosolic CK2α/α’ staining in tumor samples decreased slightly although the differences were not statistically significant (Figure 2B, left panel). These results would agree with a specific nuclear accumulation of CK2α/α’ in ccRCC, as reported recently [27]. It is worth to mention that the results in Figure 2B show the expression levels of both catalytic subunits in two different cellular compartments using the H-score method, while in the western blot assays shown in Figure 1 CK2α and CK2α’ levels were analyzed separately but in a total cellular lysate. Therefore, the augmented values for the CK2α’ subunit observed in Figure 1 might relate to the contribution of the augmented expression of this subunit in the nuclei.

In contrast to CK2α/α’, nuclear CK2β staining showed only moderate increases in tumor samples whereas cytosolic CK2β showed a significant decline in G1/G2 that was particularly marked in G3/G4 samples (Figure 2C, left panel). When the samples were grouped according to their tumor stage (pT1/pT2 and pT3/pT4) the trend of changes in CK2α/α’ mirrored that detected when grouped by grade. A similar observation was evident concerning CK2β levels (Figure 2B and 2C).

The behavior of CK2β subunit in tumors has been less explored, in contrast to the well characterized increases in CK2 catalytic subunits (CK2α/α’) in diverse types of carcinomas. A critical point addressed in our study was if the profile of CK2β changes mirrored or not that of CK2 catalytic subunits as indicative of potential changes in tetrameric CK2 form. Our results would agree with increases in CK2 holoenzyme in the initial stages of ccRCC but not in the more advanced tumors, regardless of the changes in the individual CK2α or CK2α’ subunits, since both were found increased at the initial and advanced ccRCC tumor stages.

### Downregulation of CK2β in HK-2 and 786-O renal cell lines decreases specifically the activity of CK2 holoenzyme

Human renal cell line 786-O is a widely used model of human ccRCC cells, whereas HK-2 cells are a well-established model of non-malignant human kidney PTC cells. Both cell lines express the two CK2 catalytic subunits (CK2α, and CK2α’) as well as the regulatory CK2β (Supplementary Figure 3). However, the levels of CK2α and CK2α’ were slightly higher (1.4 to 1.7-fold) in 786-O cells than in HK-2 cells whereas CK2β levels were similar. Increases in the ratios between CK2 catalytic and regulatory subunits have also been observed in metastatic breast cancer cell lines as compared with non-malignant mammary gland epithelial cells [36].

**Figure 3:**
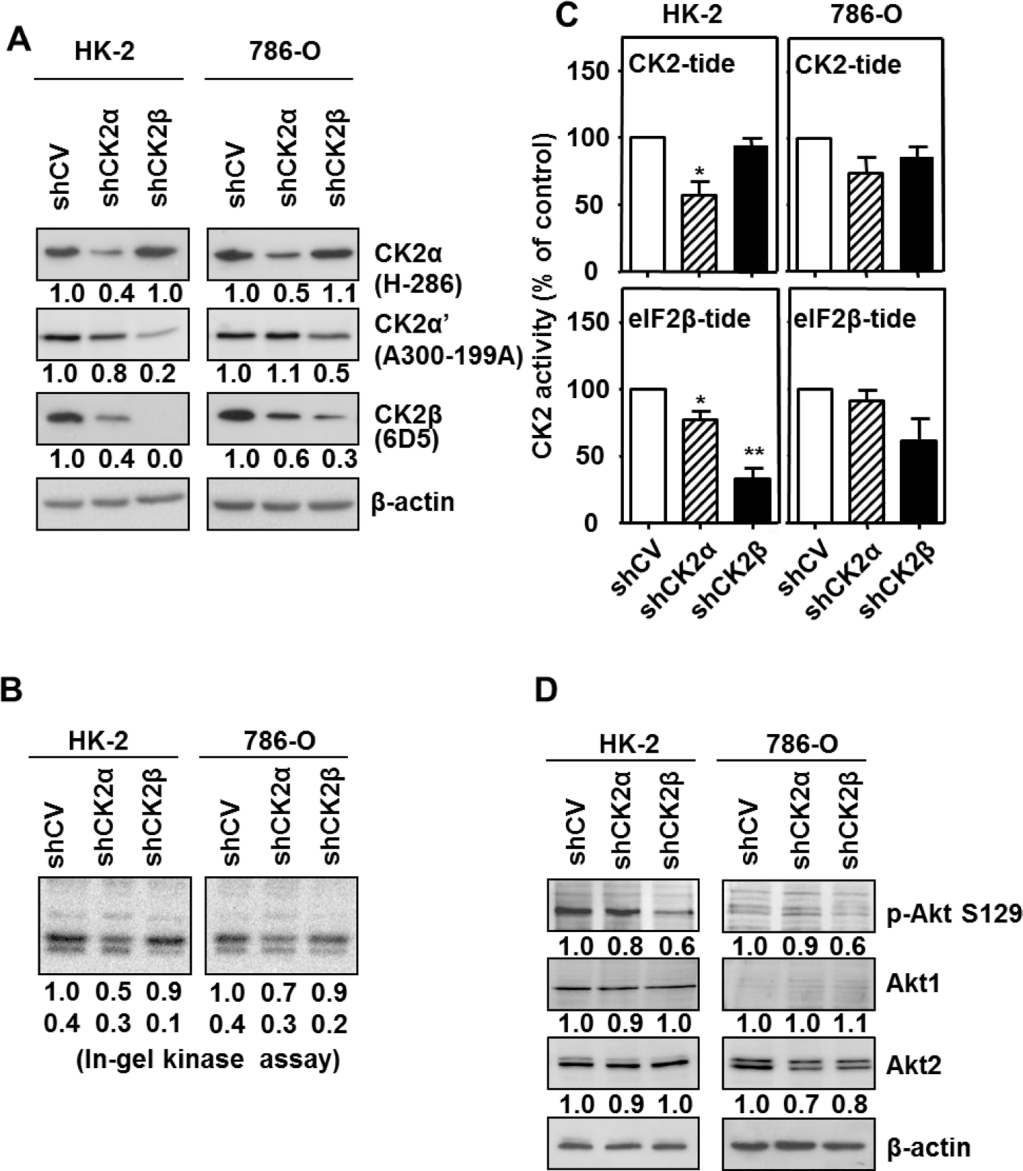
Effects of CK2α or CK2β-downregulation on CK2 subunits levels and CK2 activity in HK-2 and 786-O cell lines (**A**) Western blot analysis of CK2α, CK2α’ and CK2β expression levels in HK-2 and 786-O cells lines silenced for either CK2α or CK2β subunits. The type of antibody used to detect each CK2 subunit is indicated in parenthesis. Values below each band correspond to the relative subunit expression level respect to their corresponding control cell line (either HK-2 shCV or 786-O shCV). (**B**) In-gel CK2 activity assay with β-casein as substrate. (**C**) CK2 activity of silenced cell lines using either the CK2-tide (phosphorylated by the holoenzyme and catalytic subunits) or the eIF2β-tide (phosphorylated by CK2 holoenzyme). Data are represented as mean +/- SEM. (**D**) Effects of CK2α or CK2β-downregulation on Akt1 phosphorylation at Ser129. Akt1 phosphorylation at its Ser129 (p-Akt S129) and total Akt1 and Akt2 levels were detected in cell extracts from HK-2 and 786-O cells lines silenced for either CK2α or CK2β subunits by western blot using specific antibodies. Values below each band correspond to the relative protein expression level respect to their corresponding control cell line (HK-2 shCV and 786-O shCV).

Lentiviral transduction of shRNA-CK2α caused the stable silencing of CK2α to about 40–50% of the initial value both in 786-O and HK-2 cell lines, as compared with their respective empty-vector transduced cells (shCV) (Figure 3A). CK2α-silencing had minor consequences in CK2α’ levels but was accompanied with a decrease in CK2β levels to about 60% in 786-O and to 40% in HK-2 cells. Decreases in CK2β levels in response to CK2α-silencing have also been reported previously in other cell types due to the instability of CK2β once free [37, 38]. Lentiviral transduction of shRNA-CK2β provoked a decrease in CK2β to about 30% of the initial value in 786-O and to less than 10% in HK-2 cells. As observed in other cell lines [37, 38], CK2β-silencing caused a decrease in CK2α’. In contrast, CK2α levels were essentially unaffected in CK2β-silenced cells.

The consequences of the changes in the cell levels of CK2 catalytic/regulatory subunits on its activity was first determined in cell extracts using an in-gel CK2 activity assay with β-casein as substrate. As shown in Figure 3B, the extent of β-casein phosphorylation detected in these assays correlated with the amount of catalytic subunit, either CK2α or CK2α’ detected by western blot. We have previously shown that the standard CK2 peptide (CK2-tide) is a substrate for both CK2 holoenzyme and free catalytic subunits whereas the phosphorylation of the eIF2β derived peptide (eIF2β-tide) shows an absolute requirement for CK2β [39]. CK2 activity assays in crude cell extracts from both the HK-2 and the 786-O cell lines showed that the negative consequences of CK2α-silencing were more marked than those of CK2β-silencing when using the CK2-tide whereas those caused by CK2β-silencing were more evident on eIF2β-tide (Figure 3C). The combined data obtained on CK2 subunits content and activity assays indicate that CK2 present in CK2α-downregulated cells would correspond to CK2 holoenzyme, either tetramers or other higher rank structures, whereas the CK2β-downregulated cells, in particular the HK-2/shCK2β cells, would contain low amounts of CK2 holoenzyme which would be responsible for the activity on the eIF2β-tide.

We also verified the reduction of CK2 activity in the downregulated cells by means of the endogenous substrate Akt1 Ser129 [40]. Its phosphorylation was reduced in HK-2 cells by down-regulation of CK2 subunits, especially CK2β (Figure 3D), suggesting that it requires the holoenzyme form of CK2. This agrees with that observed recently in C2C12 myoblast cell line after knocking out CK2 subunits [38]. In the case of 786-O cells, the mainly expressed Akt isoform is Akt2, which is not phosphorylated by CK2 [41], thus phosphorylation of Akt1 at Ser129 was hardly appreciable; nevertheless it was also reduced by down-regulation of CK2 subunits, especially of CK2β.

### CK2α- or CK2β-downregulation alters the expression of EMT markers in HK-2 and 786-O cells

**Figure 4:**
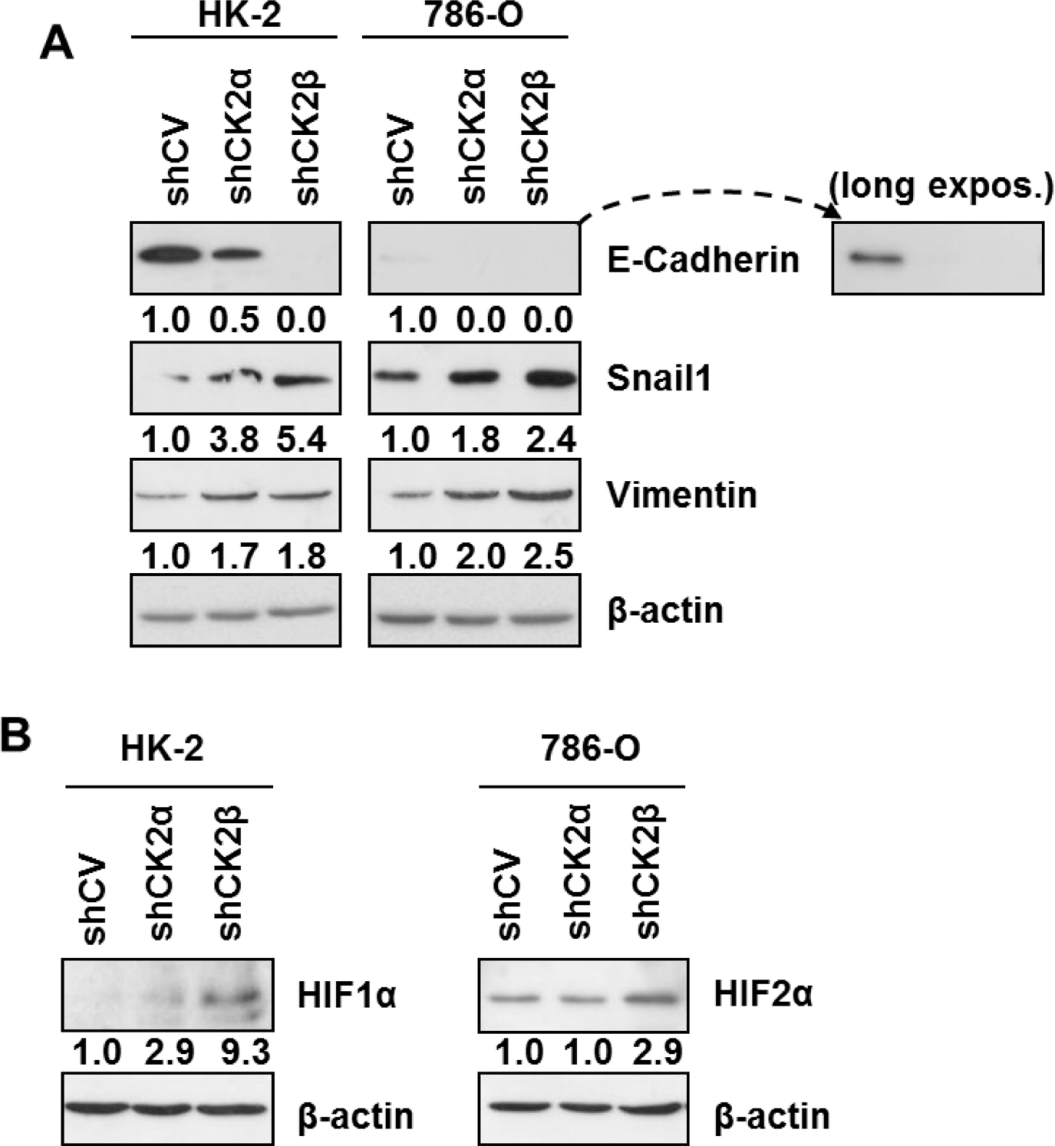
Expression of EMT markers and HIFα in HK-2 and 786-O cells silenced for CK2 subunits (**A**) Western blot analysis of the epithelial (E-cadherin) and mesenchymal markers (Vimentin, Snail1) in HK-2 and 786-O cell lines silenced for either CK2α or CK2β subunits. Values below each band represent the relative protein expression level respect to their corresponding control cell line (HK-2 or 786-O shCV). (**B**) Effect of CK2α or CK2β-downregulation on the relative expression of HIF1α in HK-2 cells and of HIF2α in 786-O cells. Values below each band represent the relative protein expression level respect to their corresponding control cell line (HK-2 or 786-O shCV).

Recent reports have shown CK2 holoenzyme activity contributes to maintaining a normal epithelial morphology in breast cell lines, as CK2β silencing induced an epithelial-to-mesenchymal transition (EMT) phenotype [24, 36, 42] associated with decreases in E-cadherin and increases in snail1 and vimentin levels. We decided to check if the decreases in CK2 holoenzyme caused by downregulation of either CK2α or CK2β altered the protein levels of these EMT markers in HK-2 and 786-O renal cell lines with respect to their corresponding controls (shCV). Control HK-2 cells expressed significant levels of E-cadherin whereas 786-O cells expressed very low levels, which were only detectable after long exposure of the blot (Figure 4A). In both cell lines, silencing of CK2α, and in particular of CK2β, caused a marked decrease in E-cadherin. Snail1 is a well-known repressor of E-cadherin expression whose stability in MCF10A cells is controlled through hierarchical phosphorylation by CK2 and GSK3β, a process that depends strongly on CK2β levels [24]. In agreement with this, CK2β silencing caused an increase in snail1 protein levels in 786-O and in HK-2 cells although both basal and stimulated levels were more robust in 786-O cells (Figure 4A). Intermediate increases in snail1 were detected in both cell lines after CK2α-silencing what agrees with the intermediate decreases in E-cadherin levels detected after silencing this subunit. Vimentin is a positive EMT marker whose expression is enhanced by snail1 [43]. Control HK-2 and 786-O cells expressed detectable levels of vimentin, which increased in the CK2-silenced cells.

The most dramatic effects on these EMT markers were exerted by CK2β down-regulation and they were particularly evident on E-cadherin and snail1 in the HK-2 cell line. The effect of rescuing CK2β levels in HK-2/shCK2β cells was then studied in order to confirm the specificity of the effects on E-cadherin and snail1. To this purpose, HK-2 cells were transfected with a vector containing a synthetic CK2β optimized coding sequence carrying base mutations that would weaken its recognition by the shCK2β without introducing any mutation into the CK2β expressed protein. Overexpression of CK2β in control HK-2/shCV cells did not alter snail1 and E-cadherin levels, whereas an increase in E-cadherin and a decrease in snail1 levels were detected in the transfected HK-2/shCK2β cells (Supplementary Figure 4). These results agree with those reported previously in MCF10A cells [24].

Previous reports have shown that HIFα is upregulated in ccRCC and could be involved in the induction of an EMT phenotype in renal cell lines [44, 45]. On the other hand, CK2 activity has been shown to affect HIF-1α transcriptional activity and to stabilize HIF-1α protein [15, 16, 46]. Therefore we decided to explore the potential effects of CK2 holoenzyme downregulation through CK2α- or CK2β-silencing on HIFα expression in HK-2 and 786-O cells under the normoxic conditions used throughout our studies. HIF-1α levels, the sole isoform of HIFα expressed in HK-2 cells, were very low in control cells, did not essentially vary in CK2α-silenced cells but tended to increase in CK2β-silenced HK-2 cells (Figure 4B). On the other hand, 786-O cells are known to express only the HIF-2α isoform. HIF-2α levels did not change in CK2α-silenced cells but were significantly higher in CK2β-silenced 786-O cells.

### CK2β-downregulation affects STAT3 phosphorylation in HK-2 and 786-O cells

STAT3 phosphorylation has been shown to affect HIF-2α levels in 786-O cells and HIF-1α and HIF-2α in Caki-1 renal cancer cells [11, 47], and STAT3 phosphorylation at Tyr705 and Ser727 residues is increased in ccRCC tumors [13]. Previous reports have shown that exposure of cells to CK2 chemical inhibitors (which block the activity of both CK2 holoenzyme and free catalytic subunits) TBB or CX-4945 diminished p-STAT3 Tyr705 [17, 37] as well as p-STAT3 Ser727 phosphorylation in response to different stimuli [18, 19]. More recently, recombinant human CK2 holoenzyme has been shown to phosphorylate ‘*in vitro*’ recombinant human STAT3 on Ser727 but the potential role of CK2β in regulating STAT3 phosphorylation, in particular at Ser727, was not evaluated [48]. Hence, we decided to determine if CK2β affected p-STAT3 Tyr705 and p-STAT3 Ser727 levels in the cultured renal cells.

**Figure 5:**
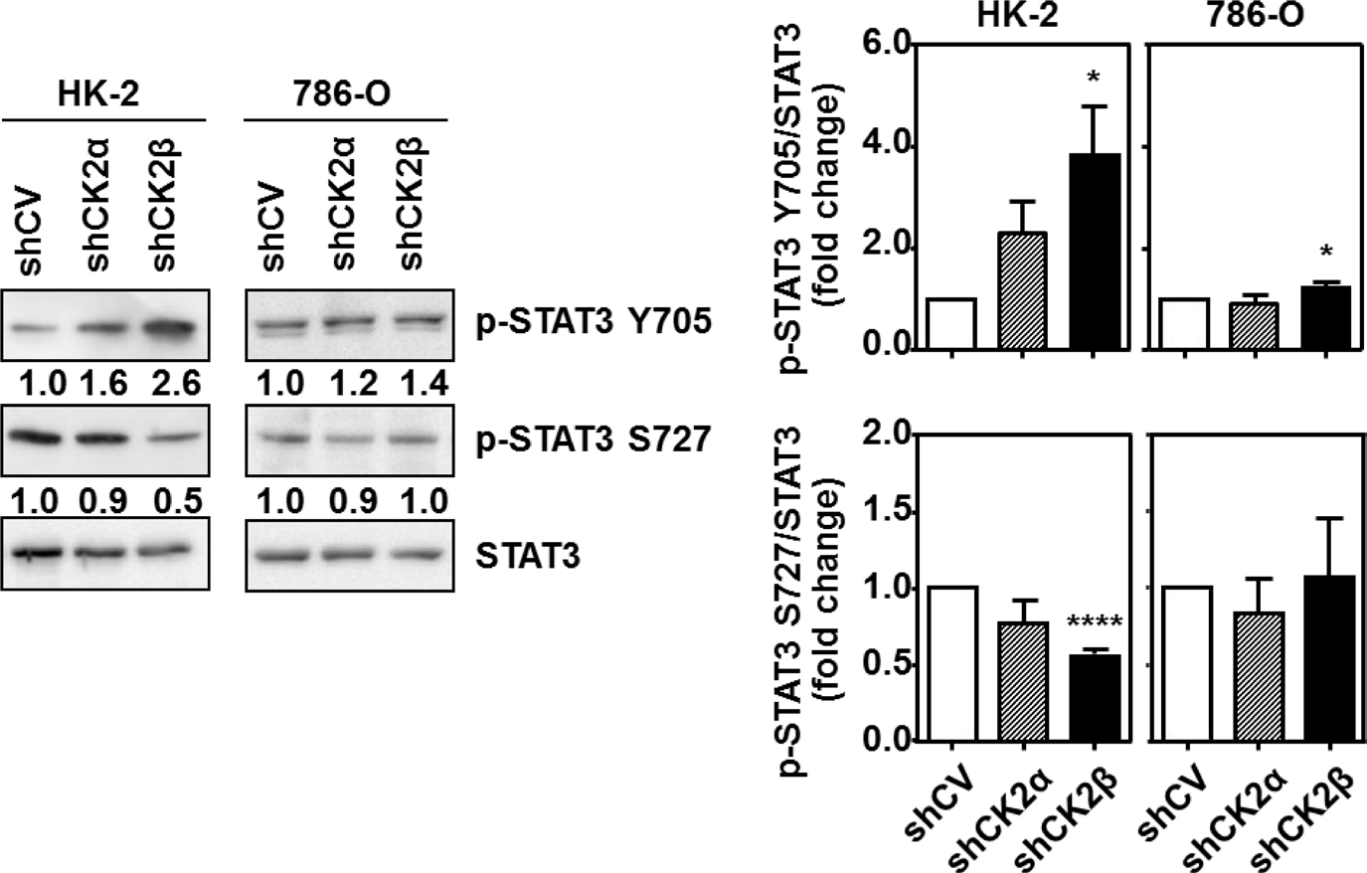
Effects of CK2α or CK2β-downregulation on STAT3 phosphorylation in HK-2 and 786-O cells STAT3 phosphorylation at either Tyr705 (p-STAT3 Y705) or Ser727 (p-STAT3 S727) and total STAT3 levels were detected in cell extracts from HK-2 and 786-O cells lines silenced for either CK2α or CK2β subunits by western blot using specific antibodies. The plots indicate the mean values of the densitometries of the p-STAT3 bands relative to the total STAT3 levels obtained in four different experiments. Data are represented as mean ± SEM. Values were analyzed by Student’s *t*-test. (^*^) and (^****^) denote *p <* 0.05 and *p <* 0.0001, respectively.

In HK-2 cells p-STAT3 Tyr705 levels tended to increase in CK2α-silenced cells and in particular in CK2β-silenced cells (Figure 5). In contrast p-STAT3 Ser727 levels tended to decrease after CK2α silencing and decreased significantly after CK2β silencing. Downregulation of CK2α did not significantly alter STAT3 phosphorylation at Tyr705 in 786-O cells whereas CK2β downregulation caused a small but significant increase in p-STAT3 Tyr705. Interestingly, in this cell line downregulation of CK2α also caused a small decrease in p-STAT3 Ser727 levels, whereas downregulation of CK2β did not decrease them. These results suggest that, unlike the HK-2 cells, the tumorigenic 786-O cells do not depend on CK2β for sustaining p-STAT3 Ser727 levels.

### Cytosolic CK2β levels help to refine the prognostic value of pSer727-STAT3 in ccRCC patients

When the data from the ccRCC TMAs were grouped by Fuhrman grade or stage no differences were found for the CK2α/α’ subunits, either nuclear or cytosolic, among groups but a clear tendency was observed for high grade and high stage tumors expressing low levels of cytosolic CK2β subunit. In the multivariate analysis, using an H-Score threshold of cytosolic CK2β higher or equal 41 (median value), we observed a non-significant correlation (*p* = 0.703) with patient survival, which indicates that cytosolic CK2β (CK2βcyt) by itself is not useful as a prognostic biomarker of ccRCC. We have previously demonstrated that p-STAT3 Ser727 is an independent prognostic factor for ccRCC patients [13]. Since we have now observed that CK2 affects STAT3 phosphorylation we decided to evaluate whether a correlation between CK2 subunits expression and p-STAT3 Ser727 levels exists.

**Figure 6:**
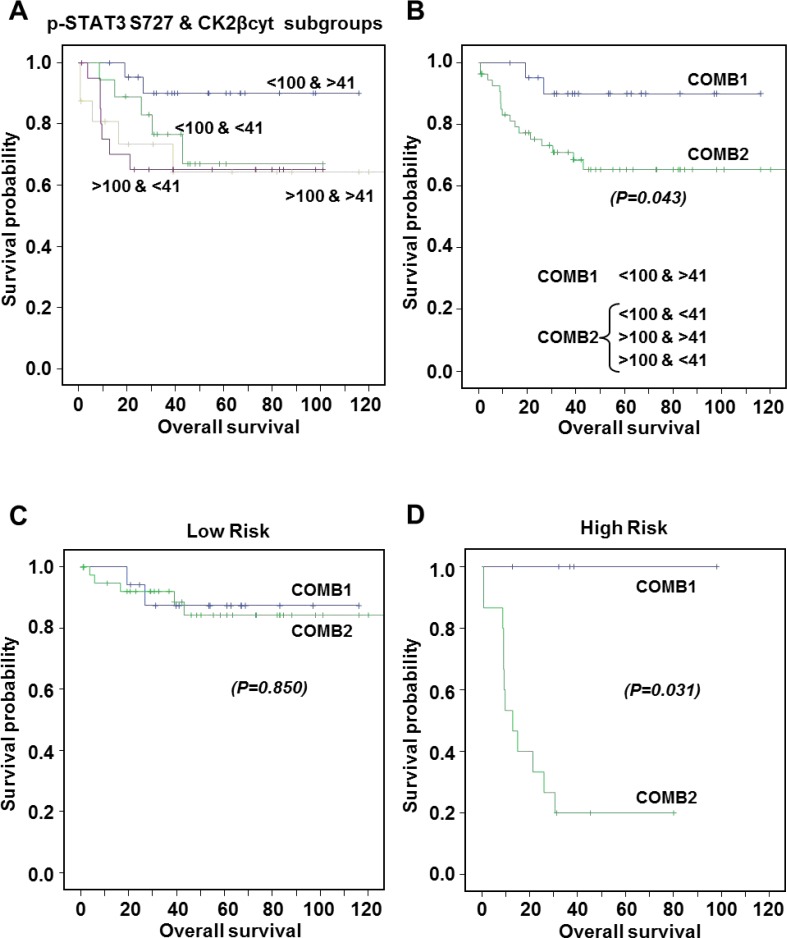
Kaplan-Meier estimates of 120-months overall survival according to combinations of p-STAT3 Ser727 and CK2βcyt expression levels (**A**) Patients exhibiting p-STAT3 S727 levels <100 and >100, correlating with good and bad prognosis, respectively [13], were subgrouped according to their CK2βcyt levels were above or below 41 (median value). Metastatic patients have been excluded. (**B**) COMB1 corresponds to patients exhibiting p-STAT3 S727 levels <100 and CK2βcyt >41. COMB2 includes the three other groups. No significant differences were observed among groups in COMB2. Statistical significant differences were observed between COMB1 and COMB2. (**C**, **D**) Kaplan-Meier estimates of 120-months overall survival according to COMB1 or COMB 2 for Low Risk (C) and High Risk (D) patients. Statistical significant differences were observed between COMB1 and COMB2 in the High Risk group.

When analyzing all patients together, we observed a negative but non-significant correlation between p-STAT3 Ser727 and cytosolic CK2β with Spearman Index of -0.057. Although the correlation was weak between both factors, we aimed to explore the impact, if any, of CK2βcyt higher or equal 41 (median value) on the survival profile of patients with p-STAT3 Ser727 levels >100 or <100, which were previously correlated with poor or good prognosis, according to patient survival, respectively [13]. We observed that the worst possible combination corresponds to p-STAT3 Ser727 >100, as reported, with no major contribution of CK2βcyt, since Kaplan Meier curves were alike when CK2βcyt was <41 or >41 (Figure 6A). Accordingly, we could say that when taking all the patients into consideration, without any kind of stratification, CK2βcyt does not add extra prognostic information to what the high levels of p-STAT3 Ser727 alone predict, regarding poor patient survival. Nevertheless, it is remarkable to observe that for patients with low p-STAT3 Ser727 levels, thereby with good prognosis according to this biomarker, those with low CK2βcyt levels (<41) (Figure 6A) exhibited a similar survival rate than patients with high pSer727-STAT3 levels. When patient represented in Figure 6A corresponding to the pSer727-STAT3>100/CK2βcyt>41; pSer727-STAT3>100/CK2βcyt<41 and p-STAT3 Ser727<100/CK2βcyt<41 groups were combined (COMB2), significant statistical differences (*p* = 0.043) appeared with the p-STAT3 Ser727<100/ CK2βcyt>41 patient group (COMB1) (Figure 6B). Accordingly, we can say that CK2βcyt refines the prognostic value of p-STAT3 Ser727 in patients with apparent good prognosis according to their low p-STAT3 Ser727 levels.

The prognostic value of p-STAT3 Ser727 was highly significant and even more pronounced in high-risk patients, were survival after 10-year follow-up was HR = 3.32 (95%IC 1.26–8.71) *p* = 0.014, by a multivariate analyses [13]. When analyzing the prognostic value of the combined biomarkers in separated high- and low-risk groups of patients, we observed that while in the low-risk patients no significant differences (*p* = 0.850) were observed between COMB1 and COMB2 markers (Figure 6C), a striking significant difference (*p* = 0.031) was observed for patients in the high-risk group according to COMB1 or COMB2 markers (Figure 6D). Kaplan Meier curves indicated that patients with p-STAT3 Ser727<100/CK2βcyt>41 (COMB1) were free of disease with no events in 10 years post-surgery, while those with p-STAT3 Ser727<100/CK2βcyt<41 were falling to profiles similar to patients with high p-STAT3 Ser727 levels. To sum up, our results indicate that CK2βcyt can refine the prognostic value of p-STAT3 Ser727 in patients within the high risk group, and that patients with high CK2βcyt have better prognosis than those with low CK2βcyt levels when p-STAT3 Ser727 are low (<100).

## DISCUSSION

Different reports have shown a link between the expression of CK2 catalytic subunits (CK2α/α’) and diverse types of carcinomas [26–32, 49, 50], The results of our study agree with an increase in nuclear CK2 catalytic subunits content in ccRCC, as previously reported in other human cancers [27, 28, 32, 49]. On the other hand, studies on the status of the regulatory CK2β in cancer are rather scarce. Our data on western blot analysis partially agree with those of a previous report, published two decades ago [25], since we detected increased total CK2β levels in the G1/G2 tumor samples. However, this does not hold for the ccRCC samples corresponding to more advanced tumors (G3/G4 and pT3), which showed decreases in total CK2β levels when analyzed by western blot or more specifically in cytosolic CK2β when analyzed by immunohistochemistry. Increased ratios between catalytic and regulatory subunits, due in part to decreased levels of the regulatory CK2β subunit, has also been detected previously in five out of a set of fifteen human renal cell carcinoma cell samples analyzed by western blot [26]. Moreover, a marked downregulation of CK2β mRNA expression in all tumor samples compared with normal renal tissue was detected in that study [24] suggesting an important posttranscriptional regulation of CK2 subunits in RCC. In contrast to this, a more recent study [27] has shown that CK2β mRNA expression was slightly higher in ccRCC than in renal cortex but no correlation was observed between CK2β mRNA expression and clinicopathological factors. However, the protein levels of CK2β were not determined in these studies. Our results show low levels of cytosolic CK2β subunit in high grade and high stage tumors, as discussed later in more detail.

The asymmetric changes in CK2 subunits detected in the ccRCC samples would reflect alterations in the ratio of CK2 catalytic subunits incorporated into CK2 holoenzyme, what could alter the targeting of a subset of CK2 specific substrates [51, 52]. As reported previously for normal human breast epithelial cells (MCF10A cells) [24], depletion of CK2β in HK-2 and 786-O cells altered the expression of EMT markers, decreasing E-cadherin and increasing snail1 and vimentin levels. These effects, together with the specific decrease in CK2 activity on the eIF2β-tide, without altering that on the CK2-tide, would support a marked specific decrease in CK2 holoenzyme in the silenced cells. On the other hand, down-regulation of CK2α induced a partial change in these parameters, indicative of intermediate decreases in CK2 holoenzyme. Altogether, our results suggest that the decreases in CK2β might contribute to the EMT in RCC tumors, a process suggested to play a significant role in disease recurrence, invasion and metastasis [6].

It is well known that ccRCC are highly vascularized tumors. Accordingly, angiogenesis is considered fundamental to ccRCC pathogenesis and HIFα is upregulated in these tumors [8, 9]. Protein kinase CK2 has been shown to affect HIF-1α transcriptional activity [46] and to stabilize HIF-1α protein either by phosphorylation or destabilization of VHL protein [15, 16] or through the histone deacetylase (HDAC)-mediated pVHL down-regulation pathway [14]. Interestingly, free recombinant human CK2α is able to phosphorylate the N-terminal acidic domain of pVHL [53] and CK2 catalytic subunits, rather than CK2 holoenzyme, were found important for triggering the HDAC-mediated pathway in HeLa cells [14]. Nevertheless, it is interesting to remark that in the present study the effects of CK2-silencing on HIFα levels were detected under normoxic conditions and HIF-2α increased in the VHL-deficient 786-O cells, what indicates that these effects are exerted, at least in part, through VHL-independent mechanisms.

Activator of transcription STAT3 is a potent regulator of HIF-1α and HIF-2α expression [11] and STAT3 phosphorylation at Tyr705 and Ser727 residues is increased in ccRCC tumors [13]. Previous reports have shown that CK2 modulates STAT3 activation since TBB and CX-4945 (two CK2 chemical inhibitors that block the activity of both CK2 holoenzyme and free catalytic subunits) diminished STAT3 phosphorylation on Tyr705 [17, 37] and Ser727 [18, 19] residues triggered in response to different stimuli in cultured cells. STAT3 phosphorylation at Tyr705 is catalyzed by Jak1 or Jak2 kinases and both are activated by binding to CK2 [17]. Our results would point to CK2α rather than CK2 holoenzyme as the positive regulator of Tyr705-STAT3 phosphorylation since it increased upon CK2β downregulation both in HK-2 and 786-O cell lines. It must be emphasized that our present study is based on downregulation CK2β which did not result in decreases on CK2 activity measured with the CK2-standard peptide in contrast to that caused by the CK2 chemical inhibitors used in most of other previous studies.

As far as STAT3 phosphorylation on Ser727, overexpression of CK2 has been shown to decrease it in C6 glioma cells through a mechanism involving CK2 activation of protein phosphatase PP2A [54]. On the other hand, a recent report has shown that recombinant human CK2 holoenzyme is able to directly phosphorylate ‘*in vitro*’ recombinant human STAT3, but the potential requirement of CK2β for this phosphorylation was not evaluated [48]. These authors also indicate that STAT3 phosphorylation on Ser727 in chronic lymphocytic leukemia (CLL) is more elaborate since it requires the assembly of a CK2/CD5/B-Cell Linker Protein (BLNK)/STAT3 complex [48]. This may underlay a unique targeting mechanism accounting for the phosphorylation of a residue, STAT3 Ser727, which is devoid of the canonical CK2 consensus. Interestingly, CD5 is known to interact with CK2 through contacts between the cytoplasmic domain of CD5 and the N-terminal region of CK2β [55]. The existence of protein complexes acting as a bridge between CK2 and STAT3 in renal cells is unknown. However, our results show that CK2β is needed for the constitutive phosphorylation of STAT3 on Ser727 in HK-2 cells, but this does not hold for 786-O cells, whose constitutive pSer727-STAT3 levels do not seem to depend on CK2β. Although the underlying reason is unknown, it clearly shows a different behavior between the non-tumorigenic HK-2 and the tumorigenic 786-O cell lines concerning the potential mechanisms involved in STAT3 phosphorylation at Ser727.

We have recently observed that STAT3 phosphorylation both at Tyr705 and Ser727 residues is increased in ccRCC tumor samples, and that Ser727 is an independent prognostic factor in ccRCC [13]. In the present study we observed that the combination of p-STAT3 Ser727 and either nuclear or cytosolic CK2α/α’ did not improve the prognostic values of p-STAT3 Ser727. No significant correlation was found between cytosolic CK2β (CK2βcyt) levels with patient survival, which indicates that CK2βcyt by itself is not useful as a prognostic biomarker. However, we have observed that CK2βcyt levels are instrumental to refine prognosis of patients with low p-STAT3 Ser727. In this group of patients, CK2βcyt>41 discriminates patients free of disease for a period of 10 years from those with CK2βcyt<41, which behave as the patients with p-STAT3 Ser727 levels over 100. Therefore, CK2βcyt is useful to fully assess the prognosis of patients with low p-STAT3 Ser727, because discriminates well among patients with different prognosis requiring different clinical follow-up that would be otherwise treated equally.

## MATERIALS AND METHODS

### TMAs and human biopsies samples

Tissue Microarrays (TMAs) were provided by the Department of Pathology, Vall d’Hebron Hospital, Barcelona, Spain. TMAs were constructed using the Advanced Tissue Arrayer (Chemicon International) as has been detailed in previous reports [13, 56]. 98 samples of renal cell carcinomas from patients subjected to a nephrectomy were included in those TMA to evaluate the expression of different biomarkers by immunohistochemistry (IHC). 21 samples from patients affected for ccRCC, which did not coincide with those of the TMA, were available for evaluating the expression of CK2 subunits by western-blot analysis.

### Immunohistochemistry

The tissue samples present in TMA were deparaffinized and an immunoperoxidase staining was used for the IHC analysis. Antigens were retrieved through a citrate buffer treatment (10 mM sodium citrate, pH 6.0) in a 95°C water bath for 20 min. The endogenous peroxidases were blocked with Peroxidase Blocking Solution (3% H_2_O_2_, 10 min) and subsequently unspecific proteins were blocked for 1h at room temperature with 10% Normal Horse Serum in PBST. The samples were incubated O/N at 4°C with anti-CK2α/α’ (H-286, sc-9030, Santa Cruz Biotechnology, which detects CK2α and CK2α’ of human origin in IHC analysis of paraffin-embedded sections at 1:50 dilution) or anti-CK2β (6D5, sc-12739 1:50 dilution, Santa Cruz Biotechnology) antibodies. After incubation, the reaction of visualization was performed with Real EnVision HRP Rabbit/Mouse Detection System (DAKO) for 40 min at room temperature using 3,3′-diaminobenzimide as a chromogen and slides finally were counterstained with haematoxylin. The expression levels of CK2 subunits were evaluated by two independent pathologists using the semi-quantitative method of immune-histo-score (H-score) based on the percentage and intensity of stained cells. Discrepancies were resolved by a concurrent re-examination by both researchers using a double-headed microscope. For each sample, nuclear and cytosolic CK2α and CK2β expression were evaluated. The intensity score was defined as: 0 = non appreciable staining in cells; 1 = weak staining; 2 = moderate staining; 3 = intense staining. [H-score = 1 × (% weak) + 2 × (% moderate) + 3 × (% intense) ranging from 0 to 300]. For each patient an H-score mean of three cores were obtained and used for subsequent statistical analysis. Snapshots were taken with a Leica DFC 500 camera coupled to a Leica DMRB microscope using the Leica Application Suite V4.3 software.

### Cell lines and transfections

Proximal tubule epithelial cell line HK2 (CRL-2190™) and human renal adenocarcinoma cell line 786-O (CRL-1932™) were obtained from the American Type Culture Collection (ATCC). HK-2 and 786-O cells were cultured in Dulbecco’s Modified Eagle’s Medium (DMEM) supplemented with 10% fetal bovine serum (FBS), 1% (v/v) L-Glutamine, 1 mM sodium pyruvate, 1% (v/v) Streptomycin and Penicillin at 37°C in an incubator.

Stably-silenced cell lines for CK2α and CK2β subunits (shCK2α and shCK2β respectively) as well as control (shCV) cell lines were generated by transduction with Sigma Mission® lentiviral Transduction Particles (Sigma-Aldrich) (shCK2α: NM_177559.2-1895s21c1; shCK2β: NM_001320.x-823s1c1; shCV: SHC202V) at a final MOI (Multiplicity of Infection) of 2 and followed by a puromycin selection (1 μg/mL) for a week. HK-2 and 786-O stably-silenced cell lines were maintained in complete DMEM medium supplemented with 1 µg/mL of puromycin in a cell incubator.

### Tissue and cell lysates and western blot analysis

Human renal carcinoma tissues were washed with cold phosphate buffered saline (PBS) and lysed in lysis buffer (50 mM Tris/HCl pH 7.7, 150 mM NaCl, 15 mM MgCl_2_, 0.4 mM EDTA, 0.5% Triton X-100, 0.5 mM DTT, 2 mM PMSF, 100 µg/ml protease inhibitors). HK-2 and 786-O cell lines extracts were obtained washing cells with PBS and lysed using the cell lysis buffer (50 mM Tris/HCl pH 7.4, 150 mM NaCl, 1 % triton-X-100, 1 mM DTT, 1 mM PMSF, 1 mM EDTA, 25 mM NaF, 0,2 mM Na_2_VO_3_, 2 mM PPi, 1 μg/mL protease inhibitors (leupeptin, benzamidin, aprotinin, pepstatin)). Protein concentrations of the extracts were determined by the colorimetric method of Bradford according to the manufacturer’s instructions (BioRad). Equal amounts of proteins were loaded in 10% SDS polyacrylamide gels (SDS-PAGE), subjected to electrophoresis and subsequently electrotransfered to polyvinylidene fluoride membranes (PVDF, Immobilion P, Millipore). Unspecific proteins were blocked in 5% non-fat dry milk in TTBS (50 mM Tris/HCl, pH 7.4, 150 mM NaCl and 0.1% Tween-20) for 1h at room temperature. Membranes were then incubated overnight with the adequate primary antibodies at 4°C, rinsed in TTBS and incubated with their respective secondary antibodies conjugated to horseradish peroxidase (HRP). The signal was generated using Lumi-light Western blotting substrate (Roche, GE) and detected by Amersham Hyperfilm ECL (GE-Healthcare) or Chemidoc MP Image System (Bio-Rad, Hercules, CA). Primary antibodies used were: anti-CK2α H-286 (sc-9030, Santa Cruz Biotechnology), anti-CK2α 1AD9 (05-1431, Millipore), anti-CK2α’ (A300-199A, Bethyl Laboratories), anti-CK2β 6D5 (sc-12739, Santa Cruz Biotechnology), anti-CK2β(h) (raised in rabbits immunized with whole human recombinant CK2β as described in [57]), anti-p-Akt1 Ser129 (ab133458, EPR6150, Abcam), anti-Akt1 (2938, C73H10, Cell Signaling), Anti-Akt2 (3063, D6G4, Cell Signaling), anti-E-Cadherin (610181, BD Biosciences), anti-Snail1 (3895, L70G2, Cell Signaling), anti-Vimentin (V6389 Sigma-Aldrich), anti-p-STAT3 Tyr705 (4113, M9C6, Cell Signaling), anti-p-STAT3 Ser727(9134, Cell Signaling), anti-STAT3 (9139, 124H6, Cell Signaling), anti-HIF-1α (ab82832, Abcam), anti-HIF-2α (ab199, Abcam), anti-β-actin (sc-47778, Santa Cruz Biotechnology). Secondary antibodies were obtained from Bio-Rad: IgG Goat Anti-Rabbit IgG (H+L)-HRP conjugate (170-6515, BioRad) and IgG Goat-Anti Mouse IgG (H+L)-HRP conjugate (170-6516, BioRad).

### Protein kinase activity assay

Protein kinase CK2 activity was determined in cell extracts using either the CK2 specific peptide (CK2-tide: RRRADDSDDDDD) or the eIF2β-derived peptide (eIF2β-tide: MSGDEMIFDPTMSKKKKKKKKP) as substrates, in the presence of phosphorylation mixture as described previously [30]. The activity exhibited by the catalytic subunits (CK2α/α’) alone was also determined by in-gel activity assays. Cell lysates were run on an 11% polyacrylamide gel electrophoresis containing β-casein (0.5 mg/ml) in the running and the stacking gel. After protein separation, proteins on the gel were renatured and then the gel were incubated with the phosphorylation medium containing 1 mM [γ^33^P]ATP [52]. The gel was stained with Comassie, dried and analyzed by autoradiography (CyclonePlus Storage Phosphor System, PerkinElmer).

### Statistical analysis

Statistical Analysis was performed using GraphPad Prism 5 program for Windows. One-way ANOVA with a Tukey’s post-hoc test was used to analyze the relationships between CK2α and CK2β expression levels in TMAs. Associations between pSTAT3 S727 and CK2β expression and clinicopathologic parameters were evaluated with the nonparametric Mann–Whitney *U* test. Disease-free survival was calculated as the date of surgery to the date of loco-regional or distant recurrence. Subsequently, Kaplan– Meier survival estimates were compared using the log-rank test. Multivariate analysis was performed using a Cox regression model to estimate the independent prognostic importance of clinicopathologic parameters. Statistical analysis was performed with the Statistical Package for Social Sciences, version 12 software (SPSS).

Student’s *t*-test was used to analyze CK2 subunits expression in ccRCC tumor samples by WB analyses as well as the cell growth and the expression and phosphorylation of the different proteins in the CK2-silenced cell. Statistical significance was set at (^*^) *p <* 0.05, (^**^) *p* <0.01, (^***^) *p <* 0.001, and (^****^) *p <* 0.0001.

## SUPPLEMENTARY MATERIALS FIGURES AND TABLE


