# Supplementary material for: Under-expression of CK2β subunit in ccRCC represents a complementary biomarker of p-STAT3 Ser727 that correlates with patient survival
Source: Oncotarget. 2017 Dec 19;9(5):5736–51. doi: 10.18632/oncotarget.23422 (PMC5814170; doi:10.18632/oncotarget.23422)
Supplement: Supplementary file 1 [file oncotarget-09-5736-s001.pdf]

Under-expression of CK2β subunit in ccRCC represents a complementary biomarker of p-STAT3 Ser727 that correlates with patient survival

SUPPLEMENTARY MATERIALS

Transient transfection of HK-2 cells

For rescue experiments human CK2β mRNA (CSNK2B) was codon-optimized and synthetized by Invitrogen GeneArt Gene Synthesis. Codon optimization tended to avoid CK2β mRNA recognition by the shRNA CK2β lentiviral system in stable silenced CK2β renal cells. Optimized CK2β mRNA was then subcloned in pCMV-HA for transient cell transfection and CK2β expression using Metafectene PRO (Biontex Laboratories, Germany).

Preparation of IgG-depleted anti-CK2β antibody (6D5)

Depletion of IgG from the anti-CK2β antibody was carried out as described previously for the obtention of serum depleted of the cancer/testis antigen CTSP-1 [1] using monoclonal anti-CK2β (6D5) antibody and His-tagged human recombinant CK2β protein obtained as indicated previously [2].

REFERENCES

1. Parmigiani RB, Bettoni F, Vibranovski MD, Lopes MH, Martins WK, Cunha IW, Soares FA, Simpson AJG, de Souza SJ, Camargo AA. Characterization of a cancer/testis (CT) antigen gene family capable of eliciting humoral response in cancer patients. Proc Natl Acad Sci USA. 2006; 103:18066–71. https://doi.org/10.1073/pnas.0608853103.
2. Llorens F, Roher N, Miró FA, Sarno S, Ruiz FX, Meggio F, Plana M, Pinna LA, Itarte E. Eukaryotic translation-initiation factor eIF2beta binds to protein kinase CK2: effects on CK2alpha activity. Biochem J. 2003; 375:623–31. https://doi.org/10.1042/BJ20030915.

Supplementary Table 1: Characteristics of the 21 ccRCC biopsies and CK2 ratios detected by Western blot

Patient	Gender	Age	Grade	Stage	CK2α	T/N Ratios	
						CK2α’	CK2β
1	M	42	G1	pT2	0.96	n.d.	0.62
2	M	74	G2	pT2	1.32	n.d.	1.44
3	M	68	G4	pT3	1.07	1.58	0.24
4	M	63	G3	pT3	0.54	7.67	0.80
5	M	37	G2	pT1	n.d.	2.90	4.55
6	M	50	G2	pT1	0.83	7.76	2.10
7	M	56	G1	pT1	0.38	4.02	2.23
8	F	n.a.	G1	pT3	1.12	n.d.	0.67
9	M	73	G2	pT1	0.55	5.35	0.49
10	M	n.a.	G1	pT1	0.76	1.28	0.63
11	M	n.a.	G1	pT1	0.94	1.43	1.62
12	M	n.a.	G1	pT2	n.d.	4.71	4.46
13	M	n.a.	G1	pT1	0.97	0.60	3.15
14	M	81	G1	pT2	1.09	2.28	1.33
15	F	54	G3	pT2	2.05	6.72	0.29
16	M	54	G2	pT2	3.43	2.88	2.35
17	M	48	G1	pT2	1.44	0.70	2.33
18	F	70	G3	pT3	4.30	3.10	0.38
19	F	53	G3	pT2	0.61	2.01	0.74
20	F	53	G2	pT2	2.07	1.81	1.22
21	F	68	G1	pT2	2.14	4.45	1.16

T, tumor; N, unaffected normal kidney counterpart.
n.a., not available; n.d., not determined.

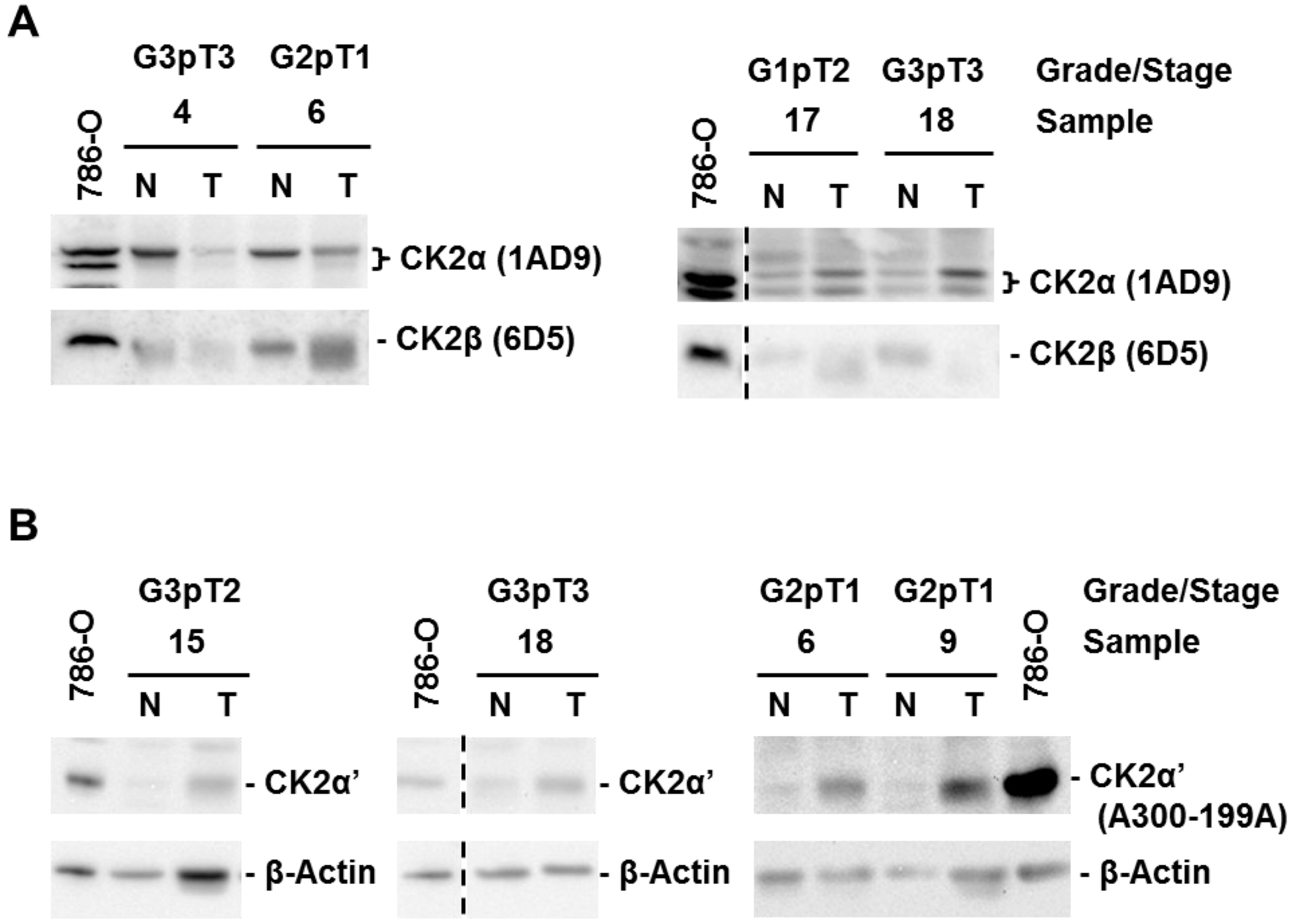

Supplementary Figure 1: Immunodetection of CK2 subunits in samples from ccRCC patients. Lysates from tumor (T) and unaffected normal (N) kidney counterpart samples from ccRCC patients were immunoblotted with antibodies to CK2α and CK2β (**A**) or to CK2α' and β-actin (**B**). 786-O cell lysates were included in the same western blots to allow the identification of the bands corresponding to CK2 subunits. Dashed vertical lines indicate that the blots included samples not pertinent to this study which were removed from gel.

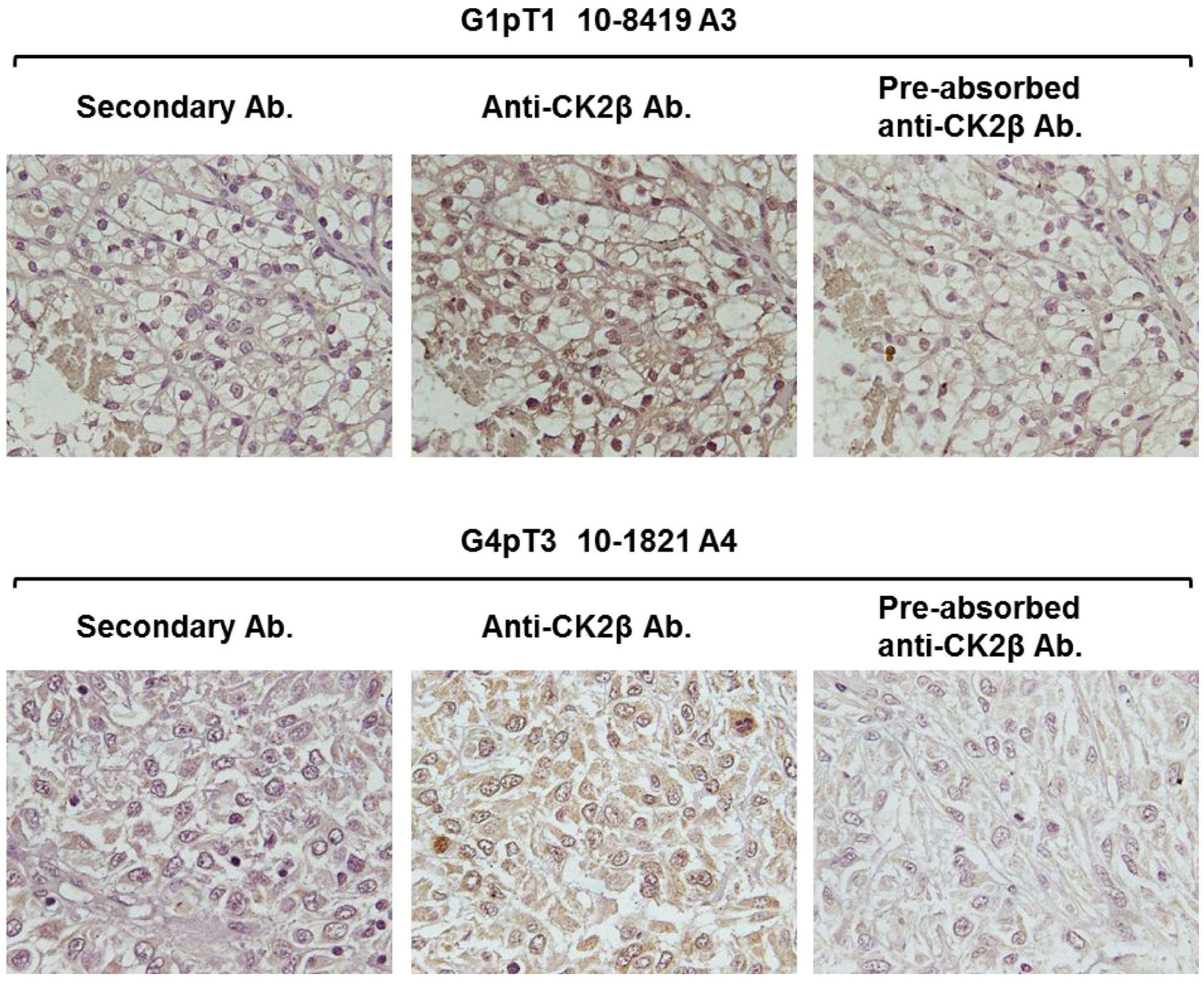

Supplementary Figure 2: Specificity of the CK2β signal in the IHC analysis of ccRCC tumors. Two tumors of different Fuhrman Grade (G) and stage (pT) were analyzed (G1pT1: 10-8419 A3 and G4pT3: 10-1821 A4) for anti-CK2β (6D5) antibody specificity using paraffin-embedded tissue sections. Tissues shown in the figure were stained with: secondary antibody (left panels), anti-CK2β antibody (middle panels) and anti-CK2β antibody pre-absorbed with recombinant CK2β protein (right panel).

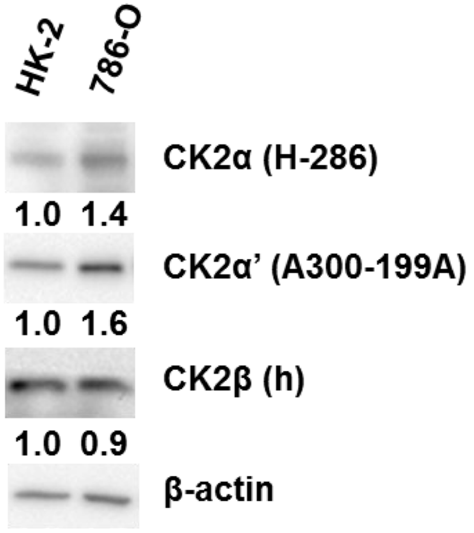

Supplementary Figure 3: CK2 subunits levels in HK-2 and 786-O cell lines. Western blot analysis of CK2α, CK2α' and CK2β expression levels in HK-2 and 786-O cells lines. The type of antibody used to detect each type of subunit is indicated in parenthesis. Values below each band in the 786-O cells denote the expression level of each subunit relative to that detected in HK-2, which was given a value of 1, once normalized to the β-actin level detected in its corresponding cell line.

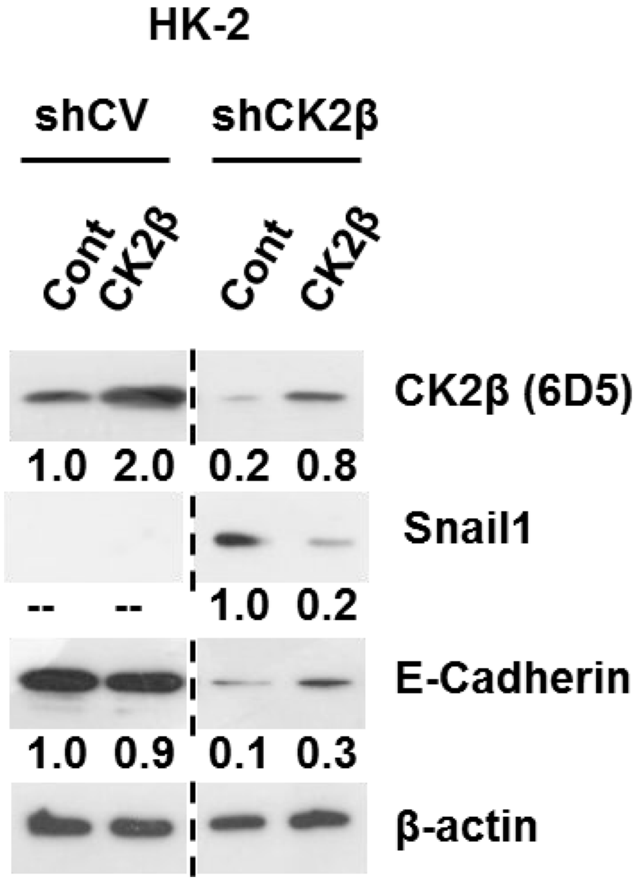

Supplementary Figure 4: Transient transfection of CK2β partially restores snail-1 and E-cadherin levels in HK-2/shCK2β cells. HK-2/shCV and HK-2/shCK2β cells were transfected with a vector containing a synthetic CK2β optimized coding sequence carrying base mutations that would weaken its recognition by the shCK2β without introducing any mutation into the CK2β expressed protein. The expression levels of CK2β, snail1, E-cadherin and actin were analyzed by western blot. Values below each band represent the relative protein expression level respect to control HK-2/shCV cell line, except for snail1 levels which are referred to those in HK-2/shCK2β cells. Dashed vertical lines indicate that the blots included samples not pertinent to this study which were removed from gel.
